# Perceived outcomes of spiritual healing and explanations - a qualitative study on the perspectives of German healers and their clients

**DOI:** 10.1186/1472-6882-14-240

**Published:** 2014-07-12

**Authors:** Michael Teut, Barbara Stöckigt, Christine Holmberg, Florian Besch, Claudia M Witt, Florian Jeserich

**Affiliations:** 1 Institute for Social Medicine, Epidemiology, and Health Economics Charité Universitätsmedizin Berlin, Luisenstr. 57, 10117 Berlin, Germany; 2 Catholic Academy The Wolfsburg, Project Medicine, Nursing, Management, Muelheim, Germany

## Abstract

**Background:**

Limited research has been conducted on contemporary spiritual healing in European countries. The aim of this article is to report how German healers and their clients experienced and perceived the outcomes of spiritual healing and which explanations they use to describe the perceived effects.

**Methods:**

Semistructured interviews and participatory observation was used to collect data from spiritual healers and their clients. Analyses were based on the methodological concept of directed qualitative content analysis. Data was analyzed using MAXQDA software, discussed and reviewed by a multidisciplinary research team consisting of medical anthropologists, medical doctors and a religious studies scholar.

**Results:**

In total 15 healers and 16 clients participated in this study, 24 interviews with healers, 20 interviews with clients and 8 participatory observations were analyzed. Healers and clients reported outcomes as positively perceived body sensations, increased well-being, positive emotions and symptomatic relief of medical complaints. Clients often described changes in their self-concepts and adapted life values. Explanations for perceived effects included connecting with transcendent sources, construction of meaning, as a result of the client-healer relationship, and as empowerment to make changes. Because the interviewed clients were recruited by the healers, a selection bias towards positive healing experiences is possible.

**Conclusion:**

We hypothesize that concepts of meaning construction, resource activation and the utilization of the clients’ expectations help to explain the data. Grounded in the emic perspective, we propose to use the following outcomes for further prospective studies: positive body sensations, changes of self-concepts and values, changes of medical symptoms and complaints. From the etic perspective, physical, emotional, social and spiritual wellbeing, sense of coherence, meaningfulness of life, empowerment, resource activation, change and symptom control should be further explored as potential outcomes.

## Background

Healers, also often called “spiritual healers”, are people who exercise above all the practice of laying on of hands and distant healing [1]. Due to globalization the techniques healers use have diversified and are derived from various spiritual and religious practices [2]. In Germany, some of these healing traditions have a long history. In the 1990s an estimated number of at least 7000 healers were practicing and treating clients, most of whom were lay healers and non-medical Complementary and Alternative Medicin (CAM) practitioners (German “Heilpraktiker”) [3]. Today the total number of German healers is unknown, but may have increased considering the positive trend of CAM usage in the last decades [4]. The German Federal Constitutional Court decided to allow spiritual healing as non-medical consulting in 2004 and made it legal for laymen to practice as long as no diseases were treated. Spiritual healing seems to be embedded in a general increase of spiritual interest by the German population, seen in the boost of esoteric and religious literature and corresponding offers on the seminar and counselling market. Globalization and the internet have also opened up new avenues to participate in a wide range of religious and spiritual systems.

Over the past two decades some quantitative studies on spiritual healers were conducted in the field of medical and psychological research [5-9]. Many of these studies aimed to investigate whether spiritual healing has a specific effect compared to control therapies. Often, the basic assumptions about the aims of treatment between biomedical researchers and healers differ: In biomedical clinical research great value has been placed on the established diagnosis (e.g. “major depression”) and its specific treatment. However, healers do not usually concentrate on a specific disease or a range of symptoms, but give attention to the “whole person”. Therefore their success might rather be attributed to spiritual and personal development than in the disappearance of specific symptoms [1,10].

Many of the quantitative studies on spiritual healing presented inconclusive and heterogeneous results. Spiritual healing itself is not well understood and remains difficult to define. Therefore, it has been argued that qualitative research strategies might help to develop better hypotheses and study designs and thus should be applied before designing further quantitative studies [9].

While there is a large body of anthropological research about indigenous healing systems in non-western countries, not much anthropological research has been conducted on contemporary European spiritual healing (see, however, [2,3,11-13]).

Addressing this lack of literature on European healers, we therefore conducted a qualitative study with German spiritual healers and their clients from 2010 to 2012. This qualitative study, performed by a multidisciplinary study team, collected data about the subjective experiences, biographies, concepts and motivations of healers and their clients. The aim of this paper is to report how healers and their clients experienced, perceived, described and explained the outcomes of spiritual healing and to generate hypotheses for future research.

## Methods

### Design

Semistructured interviews and participatory observations of healing treatments were used as qualitative research methods to collect data. The study was approved by the ethics committee of the Charité - Universitätsmedizin Berlin (02.11.2010 - EA1/238/10).

### Sample

A snowball sampling technique [14] was used for recruitment. This technique is often used in qualitative research to recruit hidden populations which are difficult for researchers to access. Healers were selected on the basis of recommendations of several German healer organizations and personal recommendations of healers or patients already included in the study. For this purpose, we established contacts with several healer organizations, which focus their membership on medical doctors, non-medical CAM practitioners (“Heilpraktiker”) and lay healers. We also visited and participated in healing events to gain access to the research field. The aim was to recruit contemporary German healers with a good reputation within their healers network. In particular we asked for addresses and contact of healers with a good reputation. We aimed to include medical doctors, non-medical CAM practitioners and lay healers. The healers were asked to arrange contact with their clients so that they could be invited by the researchers to participate in the study and as well as recommend other healers. The clients were then contacted in person, by telephone or by email and asked to participate. We paid an expense allowance of 100 Euro to the healer for participating in the interviews and 50 Euro to the client.

The following inclusion criteria applied:

Healers: 18 years of age and older, providing written informed consent. Clients: above 18 years of age, previous or current treatment by the healer, providing written informed consent.

The following exclusion criteria applied:

Healers: Lack of knowledge of the German language.

Clients: disease that makes the participation in the study impossible (e.g. being unable to speak, cognitive impairment or loss of orientation).

### Interview guideline

Based on the research questions and a literature review of publications about spiritual healing in the last 20 years a semi-structured comprehensive interview guide for healers and clients was developed by the research team. The interview guide was used to support the interviewers and allowed flexibility to vary and deepen interesting aspects. After the first interviews, the interview guideline was critically reviewed by the research team and revised accordingly. Table 1 shows the interview guideline.

**Table 1:** Interview guideline

**Questions to the healers**	**Questions to the clients**
1. Please tell me, how did you come to be a healer? Remember back: What was it like, what happened? Please describe your healing activities? Do you follow other professions besides healing? Are there healers in your family?	1. Please describe what you have experienced during the healing treatment? What did you feel like? What was your inner experience? Do you experience changes of perception in comparison to everyday consciousness? What is your experience of time?
2. Can anyone become a healer or do you need a specific gift?	2. Did you prepare yourself for the healing treatment?
3. How do people find you?	3. Where did the treatment take place? Who participates in it?
4. What do you think makes a good healer? Do you consider yourself to be a good? healer?	4. Why did you consult a healer?
5. How would you define/frame the following: disease, health and cure?	5. What did you expect from the healing treatment? Did you expect changes in your life?
6. Who visits you, how and why?	6. Can you do something yourself to help the healing process? If so, what? What role does your social surrounding play?
7. Please tell me how a healing treatment takes place? What happens? Maybe you can give me an example of a recent treatment? Do you prepare yourself for a healing treatment? Where does the healing take place? Who participates in it? Do you work together with other healers?	7. How are you, since you began the healing treatment? What exactly has changed? When did the change begin?
8. Do you work with representatives of other medical disciplines? What about conventional doctors?	8. Since you started the healing treatment, do you experience your complaints or disease differently? How do you explain this change?
9. How do you experience a healing treatment yourself? What do you feel? What was your inner experience? Do you experience changes of perception in comparison to everyday consciousness? What is your experience of time?	9. Who or what does the healing?
10. How can you tell that healing occurs? When does the healing start, when does it end?	10. If such a treatment is not curative, what else is it?
11. Who or what creates the healing?	11. How did you come to visit this healer? How did you find him? Have you been to other healers before?
12. Do you find reasons for the illnesses of your clients? If yes, at what time of the treatment? Do you communicate these reasons to your clients?	12. When was the first time you visited a healer? Remember back: What was it like, what happened? Tell me something about your life and disease history.
13. If such a treatment is not curative, what is it?	13. What do you think makes a good healer?
14. What is the follow up treatment like? What can the client do to support healing?	14. What is your definition of disease, health and cure?
15. What can you achieve through your healing treatment? What is the client’s task/role in this?	15. Would you like to add something? Have we forgotten something important?
16. In the future we have plans for studies which investigate the effect of the healing treatments . How could you most likely assess or measure the effect of a healing treatment in your opinion?	
17. Would you like to add something? Have we forgotten something important?	

### Data collection

The interviews were digitally recorded and transcribed verbatim including a pseudonymization of personal data. The text files were then added to a MAXQDA-database for the qualitative analysis. Written memos of the interviews and participatory observations of the researchers added further information on the setting, non-verbal expressions of the interviewees, as well as the researchers’ subjective experiences.

### Data analyses

Analyses were based on the methodological concept of a directed qualitative content analysis [15] by using MAXQDA software. We used a combination of deductive and inductive coding strategies: The main categories and first subcategories for coding the data were predefined by the research team according to our research questions and theexisting scientific literature. The coding of the third and fourth subcategories of data analysis followed a continuous process of extracting codes from conducted interviews and discussion among the team members. The team, consisting of three medical doctors, two medical anthropologists and one religious studies scholar with expertise in medical anthropology and health psychology, discussed the data collection and analysis every three months in a one-day research workshop. These discussions were audiotaped to document every aspect of the analysis. All coded interviews were in addition reviewed and co-coded by randomly assigned members of the research team to improve quality and validity of the analyses.

## Results

### Sample

In total 15 healers and 16 clients participated in this study. We conducted and analyzed 24 interviews with healers and 20 interviews with clients; in addition we conducted and analyzed eight participatory observations of healing sessions.

The mean age of the healers was 55 ± 7.9 (mean ± sd) years (nine males, six females; two healers did not want to provide information about their age), four healers were medical doctors, four healers were non-medical CAM practitioners (“Heilpraktiker”), another seven healers were lay practitioners. Seven healers used combinations of healing methods, nine healers practiced mainly psychic healing, eight practiced healing by hands, and seven by prayer, one healer used incantations. Of the healers that used prayer, two healers were Christian Science Practitioners. Christian Science is a faith community in which healing is considered to be a basic mission for practitioners. The other healers related to different philosophies and religions such as Christianity, Buddhism, Sufism and also Shamanism. Very often healers related to a mixture of religions and philosophies.

The mean age of the clients was 56 ± 13.8 years (13 female, three male), six clients were employed, three clients were working in healthcare, two in the media industry, one as a lawyer, one client was unemployed and four were pensioned.

The interviews lasted between 45 and 120 minutes each.

#### A. Perceived outcomes during and after healing sessions

Perceived outcomes of the healing sessions were described by all clients, but also by many healers. They can generally be assigned to the following categories:

1) Changes in sensations and feelings,

2) Changes of self concepts and values,

3) Changes of medical symptoms and complaints.

1) Changes in sensations and feelings

“Yes, in the first treatment, I’ve found so much peace and relaxation, (…) I perceived my body (…) with a pretty strong relaxation, and heat went throughout my body, which I found to be very pleasant. (…) And there’s no sense of time.” (D_H2_K1; client)

The majority of the interviewed clients reported experiencing pleasant sensations and feelings during the healing treatments, as well as in the subsequent time after the treatment. This was a consistent theme, irrespective of the type of healing treatment. Clients described multisensory perceptions with sensations of heat or light, and “energy” flowing through the body. Experiences of relaxation, inner peace, an increased feeling of harmony, inner balance and joy were most frequently reported. Some clients described and explained this perceived phenomenon as a process of “internal cleansing”. The state of consciousness during healing was described by some clients as a dreamlike condition in which the sense of time gets lost.

After the treatment, clients reported experiencing more internal strength and power, e.g. “my energy level has increased”. Some clients reported changes that started with a treatment several years back, the results continued to persist until date. Side effects or aggravations were rarely reported, these were interpreted as “temporary aggravations” in the initial process of healing.

2) Changes of self-concepts and values

“So I can let go now at all times. I totally opened myself, to my own abilities. I perceive myself to be authentic, no longer playing the role that I had played for a long time.” (C_H1_H2_K3; client)

“My whole outlook on life has changed since then. Yes, I’m actually much more conscious of my life, with my partner, with my children. Things that were important before, now no longer have any significance. Yes, it usually was the case that I felt happy when I went shopping, but this is no longer important to me.” (D_H2_K1; client)

A majority of the interviewed clients reported having changed their lives significantly after participating in healing treatments. A process of change was described by many clients that resulted in changing self-concepts, values and lifestyles. Many clients expressed that after the healing they realized how important it is to use their lifetime for a meaningful purpose instead of shopping, tv-watching, smoking or other consumer-activities. Improving family life was an important topic in talks with the healers after the treatments for many clients. Many clients reported that healing treatments helped them to focus more on social relations and to enjoy family-life and friendships. Family life was reported to have changed for the better in many cases. Some clients also embarked on new career paths, others discovered religion and spirituality, or felt more connected with transcendent sources. Some of the clients even began to act as healers themselves (see also our publication on biographical similarities between healers and their clients in: Stöckigt B, Besch F, Jeserich F, Holmberg C, Witt CM, Teut M: **Biographical similarities between healers and their clients in Germany – a qualitative study.** Submitted). Healers explained these changes as the clients’ regaining abilities to live their own lives, follow their own path and to make important decisions independently of external influences.

Clients and healers explained both that in the process of healing clients regain confidence in the meaningfulness of life, activate resources and are empowered to change. Specifically, the ability to feel and give love, to let go, to accept, to make peace and to reconcile with the personal fate, family and friends was mentioned. A process of increased self-awareness helped clients to evaluate and redefine their “life story”. The healers described this as a process in which clients become more aware of themselves and work on problems and difficulties in their actual life and biography. They take more responsibility for themselves, thus gaining control and the ability to change. Several clients described this process as an initial “shock” (becoming aware), followed by a connection to spiritual forces and experiencing a process of change.

3) Changes of medical complaints and symptoms

“Yes, I’m actually very well since then. I slept for the first time, without the usual (…) drugs.” (C_H1_H2_K5; client)

Most clients described an increase in general well-being as important outcome of the healing treatments. Ameliorations of medical symptoms and disease (e.g. symptoms of chronic headache or back pain, lymphedema, eye diseases, liver diseases, and improvements in laboratory parameters) were also described. Clients and healers, however, did not consider the medical outcomes as most important result of the healing treatments.

Most healers had the opinion that an improvement of well-being and vitality are first signs of successful healing treatments. If this happens, an improvement of medical symptoms should naturally follow.

#### B. Explanations for the perceived effects of spiritual healing

Most healers and many clients had explanations and built hypotheses on how the healing treatments might work. The described explanations can be summarized under the following categories:

1) Connecting with transcendent sources,

2) Empowering clients to change,

3) Making use of the client-healer relationship,

4) Construction of meaning.

1) Connecting with transcendent sources

“You have to understand that at the moment you feel touched by the light, you do everything right and everything is fine. (…) Love is the divine. And (…) when the divine is not there to heal, then I can not heal. (…) I let the energy flow, and I know that the energy goes to (…) where it is needed” (C_H1_H2; healer).

“What means “higher spiritual force”? This is the force that breathes in every cell of your body. (…) This is the force that moves the universe. It is the intelligence that lets the entire galaxy dance, that they do not collide together (…), the intelligence acts as above, but also works in you. Acting always and everywhere. (…) there I have to go and this is I need to find, something that has always been there. (…) Yes, it is like (…) an act of love (…), on an energetic level. You are connecting and merging with something, everything flows together.” (A_H3; healer)

Connecting clients and healers with transcendent sources to enhance healing was the most important explanation of how spiritual healing might work. Clients and healers named as transcendent sources for example “god”, “spirits”, “beings of light”, “light”, “energy”, “sacred silence”, “love”, “divine love”, “inner voice” or “the divine in oneself”. Connecting to transcendent sources in our study can be understood as a communication about an experience of transcendence (beyond oneself), as referring to something that is not in the everyday consciousness of the here and now, or something that is not experienced as a genuine part of the self [16]. The experience of connecting was described as leading to a process of “letting go”, “release” and also “merging” with the transcendent source. Healers and clients explained that connecting to transcendent sources and “letting go” is essential to start the healing process. The “self” of the client and the healer should be led and guided by the transcendent source to be transformed. Some healers described this as an “act of love”. Some healers also explained transferring love “as divine force” or “energy” in their healing to clients, which may set a transformation and healing process in motion. In other cases, love was instantly present when a connection to the transcendent sources was established. According to healers and clients the connection with transcendent sources often resulted in the perception of a “flow of energy”, and this flow should be “dynamic” and not “blocked”. Bringing “energy to flow” was often described as a central aspect of healing treatments and disease was understood as a blockage of energies. The healers described that the most important function of a healer is to act as “channel for healing, divine love and transformation”. “Cognitive thinking” of the clients was often mentioned as a hindrance in the process of healing, whereas intuition was understood as supportive and beneficial to healing.

2) Empowering clients to change

“My healing is basically (…) to make the patient aware that the responsibility lies within him, (…) my work is to lead the client to know what he needs. And then he has to do it.” (A_H3; healer)

“It is of course a great advantage that we have a selection of [clients] here who (…) want to pursue a different path, and all they need is support. They have already taken the road, that’s their energy, and all we need to do is to support them.” (C_H4; healer)

......I’ve always visualized. (..) to be healthy (..) be happy again (…) and I've always imagined exactly how it will be. And (…) that is just what happened. (C_H1_H2_K6; client)

Many healers reported that an important mechanism of healing is to enable and support clients to take more responsibility and make changes in their life. Healers reported supporting clients to change unhealthy behaviours, attitudes and lifestyles. To achieve this, healers reported utilizing pre-existing expectations and supporting the clients to activate personal resources. They guide clients to find solutions for problematic aspects of their life and to overcome psychological “blockades”. The clients’ perception of positive emotions such as “happiness” or “love” as result of healing was considered very helpful to encourage and maintain a process of change.

3) Making use of the client-healer relationship

“(I) have spoken about it with the healer, and [the psychological symptom] was subsequently gone.” (C_H1_H2_K1; client)

“And then I met [the healer] and found out that this woman is actually the first and only person I’ve ever met that really does what she says (…) and is following a very high standard of ethics (…).” (A_H2_K1; client)

Healers and clients believed that their relationship to one another supported the healing process. Clients described the healer’s empathy, his personality, his life and healing-story as a model for their individual path to healing. Trust in the healer was considered to be an essential part of the treatment. From the healer’s perspective, being empathic and giving the client the feeling of being accepted and understood was fundamental to the healing process. Taking enough time to talk about all relevant aspects of healing and to answer all the questions of the client was considered of utmost importance. Healers were often characterized by clients as role models: following the spiritual path the healer himself walked could help to change oneself. Healers inspired the clients through their personal biography, personality, their appearance and actions of faith. In most cases in the past healers themselves went through difficult times, a spiritual crisis or suffered from disease. This seemed to inspire clients to adopt similar problem solving strategies (for more details compare [15]).

4) Construction of meaning

“We all have a heart. We can access our heart, our emotions. Healing means to gain knowledge about the symptoms we are seemingly suffering from, and what they mean.” (A_H2; healer)

“So, you learn a lot about yourself. And in the broadest sense, it has a lot to do with the realization of how big this universe is and how small we humans are, and how much intelligence there is around us. And that leads strongly to the fact that you will find more in yourself!” (D_H3_K1; client)

Clients and healers reported that healing treatments helped clients to gain a new perspective on their complaints and subsequently construct new meaning in their life.

The healing sessions were most often structured as a phase of increasing the clients and healers awareness, of connecting with transcendent sources and “letting go”, and of intuitively trying to understand the meaning of symptoms and problems (e.g. “listening to your heart”). The formal healing treatment was often followed by lengthy talks between clients and the healer to interpret the experiences.

In the process of problem identification and interpretation, religious, spiritual and psychological meaning attributions were used to construct meaning. The healer emphasized that the clients “listen to their inner voice” and try to intuitively understand problems. By connecting with spiritual sources, clients could be enabled to look at their problems from a different (e.g. “higher”) perspective, learn more about themselves and understand their problems in a broader context. This leads to the construction of new meaning and subsequently problem solving strategies. This should empower clients to overcome “blockades”, make significant changes which possibly lead to relief and cure. In this sense, complaints and problems were interpreted by healers and clients as symbolic carriers of meaning. Very often they were understood as a warning system that calls for a change.

## Discussion

In the first part of the discussion we address the emic perspective of our research. In the second part, we introduce our own hypotheses and thoughts as etic perspective.

Clients and healers perceived outcomes of spiritual healing as positively experienced body sensations, positive emotions, general wellbeing and as a symptomatic relief of medical complaints. Clients reported to have changed their self-concepts, values and lifestyle. Explanations for the perceived outcomes included connecting with transcendent sources, empowerment to change, the client-healer relationship and construction of meaning. The most important explanation was “becoming a channel for transcendent sources” and “letting go”, experiencing a “flow of energy”, a “release of blockades”, “love” or “heat” which would be followed by healing. A strength of this qualitative study is the inclusion of a range of spiritual healers and their clients, different settings (rural, city), various (religious) traditions, multiple healing techniques, and professional backgrounds. Another strength is the multidisciplinary research team that enabled us to approach the data from different perspectives and utilize a large variety of scientific expertise in the process of analysis.

It was interesting to note that the answers to our interview questions between healers and clients resembled each other. This may be explained by the fact that our interviews took place after the process of healing had already occurred and healers and their clients constructed new meaning together and shared beliefs. For further studies, it would be very interesting to study this process of creating shared meanings and beliefs with a longitudinal qualitative research design, observing healing treatments over time.

A limitation of this study is a potential bias, as clients and some healers were included in this study by snowball sampling. Included healers could have thus chosen clients and other healers for the study whom they considered to be an expedient interviewee. Therefore, the reported outcomes and explanations might present a biased picture of successful healing sessions and stories of clients that improved during the healing process. We tried to minimize a potential community bias by approaching very different healer networks, which we retrospectively consider to have been successful. Nevertheless, our results may possibly reflect the more positive aspects of healing sessions. Also the fact that we observed many similarities between healers and clients might be influenced by this bias.

The subsequent research step therefore should be to include clients prospectively and analyze healing treatments qualitatively and quantitatively right from the first meeting of clients and healers. This would, in the long run, be less selective and allow for better descriptions of how the healing process develops, the experience is described, meaning is constructed and change might take place.

The described explanations for spiritual healing could be understood as interdependent mechanisms: Connecting with transcendent sources may lead to an increase in positive sensations and well-being, which is embedded in a process of creating mutual meaning between healers and clients. This might empower the client to change and help to solve problems and relieve complaints. Successful change in return may lead to positive feelings and sensations, which confirms the whole process and motivates clients to maintain the changes.

So far our analysis and discussion was closely based on the emic perspective. We now would like to introduce our etic hypotheses that should be understood as complementary interpretations of the emic explanations of our interviewees. We consider meaning construction, resource activation and utilizing the clients’ expectations as the most important hypotheses to explain the reported outcomes from an etic perspective.

### Meaning construction

Healing consists of bodily processes such as balancing, homeostatic regulation and repair, but also making sense of suffering [17]. To explain positive therapeutic responses in the context of rituals and placebo healing Brody [18] proposed a “meaning model” which consists of four elements: Positive responses may occur when 1.) the client feels listened to and attended to by the caregiver, 2.) receives an explanation of illness being consistent with his own worldview, 3.) feels care and compassion from helper or healer and 4.) experiences an increased sense of mastery or control over illness. The explanations our interviewees shared about how healing might work fit very well into this model if we consider “connecting with transcendent sources to start a healing process” and constructing meaning of symptoms as shared beliefs between healers and clients.

Transforming illness experience by cognitive reframing could play a crucial role in spiritual healing. Framing in social science refers to a schema of interpretation that individuals rely on to understand and respond to events. The choices people make in their lives are influenced by their creation of frames. The concept of cognitive reframing is based on the ideas of Ludwig Wittgenstein [19] and conceptualized by Paul Watzlawick [20]. Cognitive reframing means that a situation or an occurrence is assigned a different meaning, the situation is seen in a different context (“frame”), is differently contextualized. A popular metaphor to describe this concept is the “half filled glass”. A client may perceive a half filled glass as “nearly empty”. However, after reframing he may now perceive the glass as “half full”. Cognitive reframing may change the way people see things and create alternative ways of viewing ideas, events, situations and others. Clients participating in healing treatments start to construct new (e.g. religious or spiritual) meanings, frequently adopt the healers’ spiritual suggestions and thus reframe their individual understanding of situations. A convergence or sharing of concepts, meanings and world views between healer and clients after healing treatments can clearly be observed in our data. This exchange of experiences and ideas among healers and clients could have a moment that derives meaning, when sensations are defined as energy, love or God’s work [21,22]. A post-healing analysis could be understood as a way of ensuring a common definition of the situation and that healer and client come to some consensus over the effectiveness and meaning of the healing act [23].

Taking into consideration that cognitive reframing is a therapeutic technique used in many therapeutic systems, most prominent in psychotherapy, spiritual healing may not be that different in its mechanism, but its concepts, utilized metaphors and meanings make it particular and distinguishable from other systems. If this is true, a convergence of concepts, meanings and world views between healer and clients would be an essential condition to enable reframing and the healing process (for this hypothesis also see Jeserich 2010: 219 ff. [24]). Already in 1972 Torrey [25] described the importance of a shared world view as a cross-cultural principle of psychotherapy.

### Resource activation

The concept of resource activation may also be very helpful in understanding spiritual healing from the scientific point of view. A resource can be considered as a “source” of support, including every aspect of life that can be activated and utilized as helpful in the therapeutic sense [26,27]. The healing treatments may have enabled clients and healers to activate spiritual or transcendent resources (“connecting with transcendent sources”), personal resources (e.g. trust, motivation, positive emotions and feelings) and interpersonal resources (e.g. support of healer, family, friends). The transcendent resource seems to be of special importance here. The healer sees himself as a channel through which a transcendent force can act and strengthen the belief in the healing potential for both healer and client. The client (re-)connects to a transcendent source that can help him cope with his problems [28-30]. By focusing on positive experiences and sensations during the healing treatment, clients may start to observe positive changes and increased well-being instead of focusing on problems and negative disease experiences, thus supporting motivation through resource activation.

Our data also supports the paradigm shift that has occurred in psychology in recent decades: Overcoming illness and disease and the development of an attitude that promotes healing is associated not only with positive cognition, but also with positive emotions [31,32]. In our interviews, healers and clients talked not only of concepts and interpretations, but strikingly often emphasized associated emotions. Positive emotions such as love, trust or other positively attributed feelings could be understood as catalysts in the coping and healing processes.

### Utilizing the clients’ expectations

The utilization of clients’ pre-existing expectations was frequently described as an important instrument for healing by the healers. Today there is evidence that expectation of a therapeutic effect can lead to physiological changes and effects [33]. In placebo and psychotherapy research expectations are discussed as important effectors of healing. The term placebo and placebo effect has provoked a controversial discussion over the last decades and has no standard definition. In a pharmacological context, it is described as an inert substance with a non-specific placebo effect that accompanies any therapy and can enhance its effectiveness [34]. But this focus on neurobiological and pharmacological aspects excludes the importance of the interaction and communication of therapist and client, the positive expectations of the client, and the symbolic stimuli of a therapeutic intervention that are related to the placebo effect. All these aspects influence how an illness and its possible therapies are understood and dealt with [35,22]. Placebo responses are nowadays better understood and can be connected to neurobiological function [36,37]. According to the so-called placebo - mentalist model, the placebo effect is mainly an expectation effect showing a linear correlation of the level of expectation and the effect [33]. Most of our clients had specific expectations of healing, and the role of the healer, that included concepts but also ideas about problem solving.

### Stabilization of the sense of coherence

A helpful model to interpret our data and to integrate our findings might be the theoretical framework of the salutogenisis model of Antonovsky [27], positive experiences and emotions during the treatment could lead to a modification of the “sense of coherence” (SOC). This model was developed to show how individual cognitive and affective-motivational attitudes enable a person to mobilize appropriate resources for the preservation of health. Antonovsky defined SOC as “a global orientation that expresses the extent to which one has a pervasive, enduring though dynamic feeling of confidence that (1) the stimuli deriving from one’s internal and external environments in the course of living are structured, predictable and explicable; (2) the resources are available to one to meet the demands posed by these stimuli; and (3) these demands are challenges, worthy of investment and engagement”. The SOC has three components: (1) *comprehensibility*: a belief that things happen in an orderly and predictable fashion and a sense that you can understand events in your life and reasonably predict what will happen in the future; (2) *manageability*: a belief that you have the skills or ability, the support, the help, or the resources necessary to take care of things, and that things are manageable and within your control and (3) *meaningfulness*: a belief that things in life are interesting and a source of satisfaction, that things are really “worth it” and that there is good reason or purpose to care about what happens. Significant changes were reported by the clients of our study in all three SOC components. Comprehensibility is generated by experiences of consistency. The manageability component would receive special strengthening: it does not match any personal focus of control (“I control the action”), but, on the contrary, is associated with the confidence that there are other people and spiritual forces helping when one’s own resources are exhausted [38]. Also actively participating in decision-making and problem-solving processes strengthens this component, which was reported by our clients.

This would mean that spiritual healing may enable clients to use their resources more effectively to better deal with stressors and move on the health-disease continuum towards health. Nevertheless, it must be noted that the SOC was conceived by Antonovsky as a relatively stable global orientation. He believed that only small modifications would be possible over lifetime. However, a recent literature review [39] suggests that religious/spiritual interventions (such as mindfulness meditation) can achieve coherence- enhancing effects.

## Conclusion

The results of this study point to the following outcome parameters for future prospective quantitative studies on healing:

Outcomes derived from the emic perspective:

1) Positive body sensations

2) Changes of self-concepts and values

3) Changes of medical symptoms and complaints

Outcomes derived from the etic perspective:

1) Physical, emotional, social and spiritual wellbeing

2) Sense of coherence

3) Meaningfulness of life

4) Empowerment

5) Resource activation

6) Change

7) Symptom control

It should be remembered that spiritual healing may only fit to certain clients and problems, applying the therapeutic system to a general population might not work. In designing a prospective study on spiritual healing this should be considered. Advertising participants for a clinical trial on spiritual healing might attract completely different clients than those who naturally attend the healing treatments. Therefore, a naturalistic approach, e.g. including cohorts of routine clients of healers in prospective observational trials or comparative cohort studies might be an essential condition to further investigate spiritual healing.

## Competing interests

The authors declare that they have no competing interests.

## Authors’ contributions

All authors designed the study. BS, FB, FJ and MT collected the data. BS, FB, FJ, CH and MT analyzed the data, MT prepared the manuscript. CW and MT had the overall responsibility and received the funding. All authors were involved in interpreting the results of the analyses and critically reviewed the manuscript. The final version was approved by all authors.

## Pre-publication history

The pre-publication history for this paper can be accessed here:

http://www.biomedcentral.com/1472-6882/14/240/prepub
